# Risk factors for cognitive decline in older Chinese adults: the impact of social activity on the relationship between hearing, functional mobility, and cognition

**DOI:** 10.3389/fpubh.2024.1460941

**Published:** 2024-11-15

**Authors:** Hao Wang, Dan Chen, Daling Hu, Fangyuan Tian, Meifen Dai, Shuangyan Zhang, Liyu Jin

**Affiliations:** ^1^Department of Nursing, Sir Run Run Hospital, Nanjing Medical University, Nanjing, China; ^2^Department of Nursing, Changshu No.5 People’s Hospital, Suzhou, China; ^3^Department of Nursing, Changshu No.2 People’s Hospital, Suzhou, China

**Keywords:** self-reported hearing impairment, functional mobility impairment, social activity, cognitive function, aging

## Abstract

**Objective:**

Hearing and functional mobility impairments are recognized as risk factors for cognitive decline in older adults, yet the causal relationship underlying these associations is not well-understood. This study aims to explore whether engagement in social activities mediates the link between hearing or functional mobility impairment and cognitive decline.

**Methods:**

This cross-sectional study was carried out in two cities in Jiangsu Province, Eastern China. Participants self-reported hearing impairment and social activity engagement, whereas functional mobility impairment was assessed using the Timed Up and Go Test (TUGT). Cognitive function was evaluated through the Modified Mini-Mental State Examination (MMSE). Logistic regression analysis explored factors influencing cognitive function, and mediation analysis was conducted to examine the relationship between hearing or functional mobility impairment and cognitive decline.

**Results:**

The study included 10,217 adults aged 60 and above. Among them, 19.35% reported hearing impairment, while 40.86% failed the Timed Up and Go Test (TUGT). The Modified Mini-Mental State Examination (MMSE) indicated a 30.40% prevalence of cognitive decline. Logistic regression analysis identified significant associations of cognitive function with factors such as gender, age, education level, residency, living arrangement, hyperlipidemia, cerebrovascular disease, alcohol consumption, smoking, Activities of Daily Living (ADLs), Instrumental Activities of Daily Living (IADLs), social activity, hearing, and functional mobility (*p* < 0.01). Mediation analysis, after adjusting for confounders, showed that social activity engagement partially mediated the impact of functional mobility impairment on cognitive decline (indirect effect: −0.0947, 95% Bootstrapped CI: −0.1228, −0.0695; proportion of total effect: 11.635%, *p* < 0.01). However, no mediation effect was observed in the relationship between self-reported hearing impairment and cognitive decline.

**Conclusion:**

This study revealed that social activity engagement plays a mediating role in the relationship between functional mobility and cognitive function, but it does not significantly influence the relationship between self-reported hearing impairment and cognitive decline. These findings suggest that social activity engagement could be a crucial factor in preventing cognitive deterioration among older adults with functional mobility impairments.

## 1 Introduction

With the acceleration of global aging, the older adult population in China is expanding. The data from National Bureau of Statistics in 2023 shows that individuals aged 60 and above now number 280.04 million, comprising 19.8% of China’s total population, signaling a shift toward a moderately aged society. Aging correlates with various non-communicable diseases, such as hearing loss, functional disability, and cognitive decline (1–3). These conditions can potentially progress to more severe cognitive issues like dementia, leading to decreased quality of life and increased early mortality (4–6). Consequently, the identification of risk factors that could prevent or delay cognitive impairment is essential for health care systems.

Numerous studies, encompassing epidemiological research, systematic reviews, and meta-analyses, have established a significant association between age-related hearing loss and cognitive decline (7–12). The underlying etiological mechanisms linking the two are extensively researched but remain inconclusive. Various theoretical models have been suggested, such as the cognitive load hypothesis, common cause hypothesis, cascade hypothesis, and the overdiagnosis or harbinger hypothesis (13, 14). Under the cascade hypothesis, some studies indicate that the link between hearing impairment and cognitive decline is mediated by social engagement (15–19), while others report contrasting findings (7, 14, 20). Consequently, a definitive conclusion has not yet been reached.

Physical fitness often declines significantly with age, with extensive research underscoring reductions in muscular strength, balance, gait speed, functional mobility, and cardiorespiratory capacity in individuals over 60 years old (2). This deterioration in physical performance is consistently associated with diminished cognitive performance and accelerated cognitive decline, though the exact nature and extent of these relationships are still not fully understood (21–24). A prospective cohort study in China identified social isolation as a potential mediator in the link between physical mobility and cognitive function (25). However, the mediating role of social activity in the relationship between functional mobility impairment and cognitive decline has been less explored.

Social engagement is a critical determinant of wellbeing across all life stages (26). Substantial evidence indicated that reduced social participation was related with increased incidence of dementia (27, 28). Considering the established associations between hearing impairment, functional mobility impairment, and cognitive decline, enhancing social engagement in older adults could be a viable approach to mitigating cognitive decline. Thus, this study aimed to investigate the relationship between self-reported hearing impairment, functional mobility impairment, and cognitive decline, and to assess whether social activity plays a mediating role in these relationships.

## 2 Methods

### 2.1 Participants and basic information

The Sir Run Run Hospital is conducting a cohort study focused on monitoring older adult disability among individuals aged 60 and older. This research involved participants from diverse settings, such as communities, outpatient clinics, hospital inpatient departments, and nursing homes. Eligible participants were required to be fully conscious, capable of effective communication, and willing to participate in research activities. The present analysis utilizes baseline data collected from February 1st, 2022, to April 30th, 2023, in two cities (Nanjing and Suzhou) in Jiangsu Province. The Ethics Committee of Sir Run Run Hospital, affiliated with Nanjing Medical University, reviewed and approved our study (No. 2021-SR-047). Our study was conducted in strict compliance with the Declaration of Helsinki, and all participants signed a written informed consent form. A total of 10,217 individuals from this cohort are included in the current analysis. As part of the study protocol, all participants underwent a comprehensive standardized geriatric assessment.

Participants provided information on sociodemographic factors such as education level, marital status, alcohol consumption, and smoking habits, along with medical details including chronic conditions. Based on prior research (12, 29), our study collected extensive baseline information, including a variety of potential confounding factors. These included sex (female, male), age (as a continuous variable in years), education level (≤6 years, 7–9 years, 10–12 years, >12 years), marital status (married, single), residency (urban, rural), living arrangement (alone without a caregiver, alone with a caregiver, in a nursing home, living with family), and health-related factors such as hypertension (yes, no), diabetes (yes, no), hyperlipidemia (yes, no), cerebrovascular disease (yes, no), cardiovascular disease (yes, no), polypharmacy (none, 1–2 medications, ≥3 medications), alcohol consumption (never, used to, always), smoking status (never, used to, always), Activities of Daily Living (ADLs) (total dependence, extensive assistance, moderate assistance, limited assistance, independence), and Instrumental Activities of Daily Living (IADLs) as a continuous variable.

### 2.2 Self-reported hearing impairment

Hearing impairment was determined based on self-reported hearing difficulties. Participants responded to the question, ‘If someone is speaking in a normal voice in the room, would you be able to hear it?’ Those who answered ‘Yes’ were classified as having no self-reported hearing impairment, whereas a ‘No’ response indicated self-reported hearing impairment. Additionally, participants who reported using hearing aids were categorized as having self-reported hearing impairment.

### 2.3 Cognitive impairment

Cognitive function in this study was evaluated using the modified Chinese version of the Mini-Mental State Examination (MMSE) (30). The MMSE is extensively utilized for dementia screening, cognitive function assessment, and as a standard for determining endpoint outcomes in clinical trials. It also serves as a benchmark in the diagnostic performance and validity studies of various single-domain tests (31, 32). This scale comprises 30 items across five dimensions: orientation, registration, attention and calculation, recall, and language. Scores range from 0 to 30, with higher scores reflecting better cognitive function. The education-adjusted MMSE demonstrates enhanced diagnostic accuracy for dementia, marked by high sensitivity and specificity (33). For this study, cognitive impairment was identified using cutoff scores of 21 and 25 on the MMSE. Specifically, a score below 21 indicated cognitive impairment for participants with less than 6 years of education, and a score below 25 signified impairment for those with more than 6 years of education.

### 2.4 Functional mobility impairment

Functional mobility in this study was assessed using the Timed Up and Go Test (TUGT). The TUGT is a rapid and practical method for evaluating mobility, gait, balance, and fall risk (34). It involves timing how long it takes for a person to rise from a chair, walk three meters, turn, return to the chair, and sit down again. In our analysis, a cutoff time of 12 s was employed to categorize TUGT results into two groups: pass and fail.

### 2.5 Social activity

Social activity refers to social interactions between people, where individuals use certain tools to transmit information and exchange ideas to achieve a specific purpose. Social activities encompass various collective events aimed at maintaining interdependent and interconnected social relationships formed through interactions within a social group. For assessing social activity engagement, participants were inquired about their frequency of participation in various social activities. These activities encompassed gatherings, board games, attending events at community senior centers, or any other activities involving communication and interaction. The specific questions posed was, ‘How often do you join in social activities?’ Available response options ranged from none, 1–3 days per week, 4–6 days per week, to daily.

### 2.6 Statistical analyses

Data analysis and processing in this study were conducted using IBM SPSS Statistics version 26. Categorical variables were represented as counts and percentages, while non-normally distributed continuous variables were described using medians and interquartile ranges (IQR). Group differences were evaluated using the Mann–Whitney *U* test for non-normally distributed continuous variables and the chi-square test for categorical variables. The risk factors for cognitive decline were analyzed using binomial logistic regression. Additionally, mediation effect analysis was carried out using the SPSS macro-PROCESS program (Model 4) developed by Hayes. A *p*-value of less than 0.05 was deemed to indicate statistical significance.

## 3 Results

### 3.1 Characteristics of subjects

The analysis included 10,217 participants, with a median age of 72 years (interquartile range: 66–79), and 50.56% (5,166) of them being male. Of these participants, 30.40% (3,106) were identified with cognitive impairment, 19.35% (1,977) reported hearing impairment, and 40.86% (4,175) failed the Timed Up and Go Test (TUGT). Regarding social activity, 38.98% (3,983) reported infrequent social engagement, 31.82% (3,251) participated 1 to 3 days per week, 13.16% (1,345) engaged 4–6 days per week, and 16.03% (1,638) were involved in daily social activities. Demographic and clinical characteristics were presented in Table 1. Significant differences were observed between the two cognitive function groups (*p* < 0.001) in terms of gender, age, education level, marital status, residency, living arrangement, hypertension, hyperlipidemia, cerebrovascular disease, cardiovascular disease, polypharmacy, alcohol consumption, smoking, ADLs, IADLs, self-reported hearing impairment, TUGT, and social activity engagement.

**Table 1:** Characteristics of participants according to cognitive function.

Variables	Total (*n* = 10,217)	Cognitive function (%)		*χ*^2^	*P* ^*^
		Normal (*n* = 7,111)	Impaired (*n* = 3,106)		
Gender					
Male	5,166 (50.56)	3,726 (52.40)	1,440 (46.36)	31.506	<0.001
Female	5,051 (49.44)	3,385 (47.60)	1,666 (53.64)		
Age (years)^a^ Median (P25, P75)	72 (66, 79)	70 (65, 76)	76 (70, 83)	–	<0.001
Education					
≤6 years	6,313 (61.79)	4,200 (59.06)	2,113 (68.03)	147.162	<0.001
7–9 years	2,536 (24.82)	1774 (24.95)	762 (24.53)		
10–12 years	1,028 (10.06)	861 (12.11)	167 (5.38)		
>12 years	340 (3.33)	276 (3.88)	64 (2.06)		
Marital status					
Married	9,029 (88.37)	6,479 (91.11)	2,550 (82.10)	170.906	<0.001
Single	1,188 (11.63)	632 (8.89)	556 (17.90)		
Residency					
Urban	5,514 (53.97)	3,708 (52.14)	1806 (58.15)	31.337	<0.001
Rural	4,703 (46.03)	3,403 (47.86)	1,300 (41.85)		
Living arrangement					
Live alone without caregiver	278 (2.72)	193 (2.71)	85 (2.74)	30.622	<0.001
Live alone with caregiver	113 (1.11)	53 (0.75)	60 (1.93)		
Nursing home	842 (8.24)	567 (7.97)	275 (8.85)		
Live with family	8,984 (87.93)	6,298 (88.57)	2,686 (86.48)		
Hypertension					
Yes	6,056 (59.27)	4,039 (56.80)	2017 (64.94)	59.329	<0.001
No	4,161 (40.73)	3,072 (43.20)	1,089 (35.06)		
Diabetes					
Yes	2,250 (22.02)	1,583 (22.26)	667 (21.47)	0.779	0.377
No	7,967 (77.98)	5,528 (77.74)	2,439 (78.53)		
Hyperlipidemia					
Yes	496 (4.85)	388 (5.46)	108 (3.48)	18.333	<0.001
No	9,721 (95.15)	6,723 (94.54)	2,998 (96.52)		
Cerebrovascular disease					
Yes	1,068 (10.45)	542 (7.62)	526 (16.93)	200.303	<0.001
No	9,149 (89.55)	6,569 (92.38)	2,580 (83.07)		
Cardiovascular disease					
Yes	1,235 (12.09)	811 (11.40)	424 (13.65)	10.263	0.001
No	8,982 (87.91)	6,300 (88.60)	2,682 (86.35)		
Polypharmacy					
0	3,123 (30.57)	2,392 (33.64)	731 (23.54)	149.326	<0.001
1–2	4,760 (46.59)	3,289 (46.25)	1,471 (47.36)		
≥3	2,334 (22.84)	1,430 (20.11)	904 (29.10)		
Alcohol consumption					
Never	6,840 (66.95)	4,675 (65.74)	2,165 (69.70)	35.464	<0.001
Used to	2,503 (24.50)	1753 (24.65)	750 (24.15)		
Always	874 (8.55)	683 (9.60)	191 (6.15)		
Smoking					
Never	6,837 (66.92)	4,729 (66.50)	2,108 (67.87)	21.268	<0.001
Used to	2,243 (21.95)	1,525 (21.45)	718 (23.12)		
Always	1,137 (11.13)	857 (12.05)	280 (9.01)		
ADLs					
Total dependent	484 (4.74)	150 (2.11)	334 (10.75)	1276.008	<0.001
Extensive assistance	849 (8.31)	342 (4.81)	507 (16.32)		
Moderate assistance	1,634 (15.99)	898 (12.63)	736 (23.70)		
Limited assistance	955 (9.35)	604 (8.49)	351 (11.30)		
Independent	6,295 (61.61)	5,117 (71.96)	1,178 (37.93)		
IADLs^a^ Median (P25, P75)	20 (15, 24)	22 (18, 24)	15 (6, 19)	–	<0.001
Hearing impairment					
No	8,240 (80.65)	6,026 (84.74)	2,214 (71.28)	250.985	<0.001
Yes	1977 (19.35)	1,085 (15.26)	892 (28.72)		
TUGT					
Pass	6,042 (59.14)	4,872 (68.51)	1,170 (37.67)	851.089	<0.001
Fail	4,175 (40.86)	2,239 (31.49)	1936 (62.33)		
Social activity					
None	3,983 (38.98)	2,577 (36.24)	1,406 (45.27)	77.416	<0.001
1–3d/w	3,251 (31.82)	2,391 (33.62)	860 (27.69)		
4–6d/w	1,345 (13.16)	980 (13.78)	365 (11.75)		
Daily	1,638 (16.03)	1,163 (16.35)	475 (15.29)		
MMSE score^a^ Median (P25, P75)	25 (21, 29)	28 (25, 30)	18 (15, 20)	–	<0.001

Values are reported as *n* (%), unless otherwise indicated.

* Based on the chi-square test, unless otherwise indicated.

a Mann Whitney U test was performed for continuous variables.

### 3.2 Logistic regression analysis

Table 2 displays the outcomes of univariate logistic regression analysis concerning the risk factors for cognitive impairment. Notably, diabetes was the only factor not significantly linked to cognitive decline. In the multivariate logistic regression analysis, factors such as gender, age, education, residency, living arrangement, hyperlipidemia, cerebrovascular disease, alcohol consumption, smoking habits, ADLs, IADLs, social activity engagement, self-reported hearing impairment, and functional mobility (assessed by TUGT) emerged as independent variables associated with cognitive decline, as detailed in Table 3.

**Table 2:** Results of univariate logistic regression analysis.

Variable	Exp(B)	95% CI	*p-*value
Gender			
Female	1.000		
Male	1.273	1.170–1.386	<0.001
Age	1.079	1.074–1.085	<0.001
Education			
>12 years	1.000		
≤6 years	2.170	1.645–2.862	<0.001
7–9 years	1.852	1.393–2.463	<0.001
10–12 years	0.836	0.608–1.150	0.272
Marital status			
Single	1.000		
Married	2.235	1.977–2.527	<0.001
Residency			
Urban	1.000		
Rural	0.784	0.720–0.854	<0.001
Living arrangement			
Live with family	1.000		
Live alone without caregiver	1.033	0.797–1.338	0.808
Live alone with caregiver	2.654	1.829–3.851	<0.001
Nursing home	1.137	0.978–1.323	0.095
Hypertension			
No	1.000		
Yes	0.710	0.650–0.775	<0.001
Diabetes			
No	1.000		
Yes	1.047	0.945–1.160	0.377
Hyperlipidemia			
No	1.000		
Yes	1.602	1.289–1.991	<0.001
Cerebrovascular disease			
No	1.000		
Yes	0.405	0.356–0.460	<0.001
Cardiovascular disease			
No	1.000		
Yes	0.814	0.718–0.923	0.001
Polypharmacy			
≥3	1.000		
0	0.483	0.430–0.544	<0.001
1–2	0.707	0.638–0.785	<0.001
Alcohol consumption			
Always	1.000		
Never	1.656	1.399–1.960	<0.001
Used to	1.530	1.276–1.835	<0.001
Smoking			
Always	1.000		
Never	1.364	1.181–1.576	<0.001
Used to	1.441	1.226–1.694	<0.001
Social activity			
Daily	1.000		
No	1.336	1.179–1.514	<0.001
1–3d/w	0.881	0.772–1.005	0.059
4–6d/w	0.912	0.777–1.071	0.261
Hearing impairment			
Yes	1.000		
No	2.238	2.022–2.476	<0.001
TUGT			
Pass	1.000		
Fail	3.601	3.297–3.932	<0.001
ADLs			
Independent	1.000		
Total dependent	9.672	7.897–11.847	<0.001
Extensive assistance	6.439	5.537–7.490	<0.001
Moderate assistance	3.560	3.170–3.999	<0.001
Limited assistance	2.524	2.181–2.921	<0.001
IADLs	0.869	0.863–0.875	<0.001

**Table 3:** Results of multivariate logistic regression analysis.

Variable	Exp(B)	95% CI	*p*-value
Gender	1.180	1.030–1.353	0.017
Age	1.031	1.023–1.038	<0.001
Education			<0.001
≤6 years	1.645	1.643–1.17	0.004
7–9 years	2.406	2.403–1.704	<0.001
10–12 years	1.061	0.727–1.548	0.759
Marital status	1.173	0.973–1.416	0.095
Residency	0.678	0.604–0.759	<0.001
Living arrangement			<0.001
Live alone without caregiver	0.859	0.627–1.178	0.345
Live alone with caregiver	0.805	0.503–1.289	0.367
Nursing home	0.144	0.114–0.183	<0.001
Hypertension	0.926	0.819–1.048	0.222
Hyperlipidemia	1.417	1.086–1.844	0.010
Cerebrovascular disease	0.595	0.506–0.700	<0.001
Cardiovascular disease	1.092	0.931–1.281	0.280
Polypharmacy			0.061
0	0.977	0.820–1.161	0.790
1–2	1.123	0.981–1.284	0.092
Alcohol consumption			0.004
Never	1.171	0.938–1.456	0.159
Used to	0.884	0.702–1.110	0.293
Smoking			0.016
Never	0.738	0.600–0.908	0.004
Used to	0.830	0.675–1.021	0.077
Social activity			<0.001
None	0.566	0.484–0.663	<0.001
1–3d/w	0.512	0.437–0.600	<0.001
4–6d/w	0.745	0.621–0.895	0.002
Hearing impairment	1.542	1.36–1.745	<0.001
TUGT	1.410	1.191–1.619	<0.001
ADLs			<0.001
Total dependent	0.996	0.723–1.369	0.979
Extensive assistance	0.748	0.588–0.949	0.017
Moderate assistance	1.045	0.885–1.226	0.598
Limited assistance	1.418	1.198–1.670	<0.001
IADL	0.852	0.840–0.864	<0.001

### 3.3 Mediation analysis

**Figure 1:**
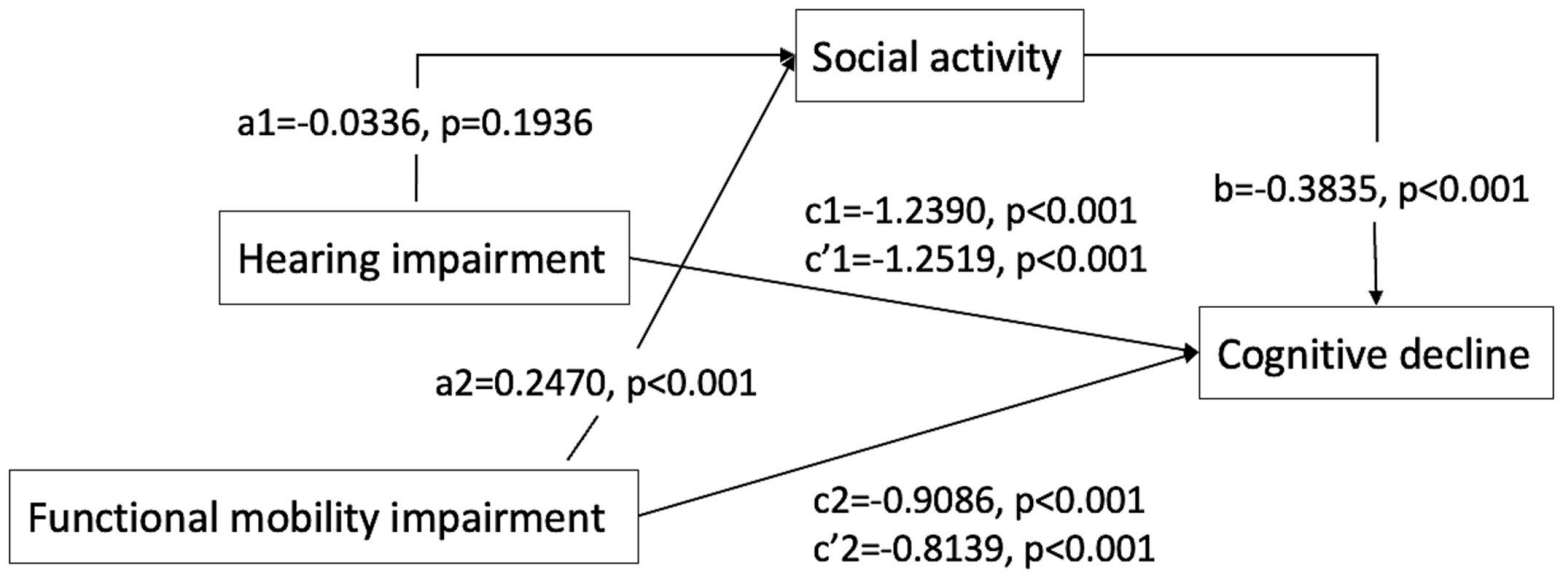
Path diagram of the mediation analysis of social activity on the relationship between hearing impairment/functional mobility impairment and cognitive decline. *c*, total effect; *c*’, direct effect; a*b, mediate effect Path1: the direct effect of hearing impairment on cognitive decline was *c*’1 [−1.2519, *p* < 0.001]. The indirect effect of hearing impairment on cognitive decline through social activity was *a*1*b (0.0129, 95% CI [−0.0056, 0.0333]), total effect was *c*1 [−1.2390, *p* < 0.001], suggesting that social activity not significantly mediates the relationship between hearing impairment and cognitive decline. Path2: the direct effect of functional mobility impairment on cognitive decline was *c*’2 [−0.8139, *p* < 0.001]. The indirect effect of functional mobility impairment on cognitive decline through social activity was *a*2*b (−0.0947, 95% CI [−0.1228, −0.0695]), total effect was *c*2 [−0.9086, *p* < 0.001], suggesting that social activity partially mediates the relationship between functional mobility impairment and cognitive decline.

The mediation analysis examining the impact of social activity on hearing, functional mobility, and cognitive function is presented in Tables 4, 5 and Figure 1. The analysis revealed that social activity significantly mediated the relationship between functional mobility and cognitive function, but it did not mediate the association between self-reported hearing impairment and cognitive function. Both the total and direct effects were significant (*p* < 0.001). In Path 1, after controlling for variables such as gender, age, education, residency, living arrangement, hyperlipidemia, cerebrovascular disease, alcohol consumption, smoking, ADLs, IADLs, and functional mobility, self-reported hearing impairment was significantly associated with cognitive decline. However, the mediation effect of social activity in this pathway was not significant. Conversely, Path 2 demonstrated that the impact of functional mobility impairment on cognitive decline was significantly mediated by social activity, after adjusting for other covariates, with an effect value of −0.0947 (95% Bootstrapped CI: −0.1228 to −0.0695).

**Table 4:** Results of mediation analysis (*n* = 10,217).

	Social activity			Cognitive function			Total effect model		
	Coeff	se	*t*	Coeff	se	*t*	Coeff	se	*t*
Constant	−1.152^**^	0.165	−6.957	18.936^**^	0.733	25.816	19.377^**^	0.734	26.383
Gender	0.223^**^	0.026	8.692	−0.697^**^	0.114	−6.117	−0.783^**^	0.114	−7.868
Age	−0.005	0.014	−0.336	−0.422^**^	0.063	−6.718	−0.420^**^	0.063	−6.664
Education	0.159^**^	0.014	11.686	1.480^**^	0.061	24.434	1.419^**^	0.060	23.496
Residency	0.244^**^	0.022	11.225	1.048^**^	0.097	10.853	0.955^**^	0.096	9.909
Living arrangement	0.037^*^	0.018	2.096	−0.589^**^	0.078	−7.532	−0.604^**^	0.078	−7.687
Hyperlipidemia	0.225^**^	0.047	4.815	−0.559^**^	0.207	−2.701	−0.646^**^	0.208	−3.111
Cerebrovascular disease	0.039	0.033	1.184	1.526^**^	0.147	10.386	1.511^**^	0.147	10.246
Alcohol consumption	0.043^*^	0.020	2.174	0.161^*^	0.088	1.827	0.144	0.088	1.633
Smoking	0.078^**^	0.020	3.919	−0.309^**^	0.088	−3.518	−0.339^**^	0.088	−3.847
ADLs	0.187^**^	0.014	13.191	0.063	0.063	0.994	−0.009	0.063	−0.141
IADLs	0.021^**^	0.003	7.764	0.385^**^	0.012	32.163	0.377^**^	0.012	31.468
Hearing impairment	−0.034	0.026	−1.292	−1.252^**^	0.115	−10.896	−1.239^**^	−0.115	−10.745
Functional motility impairment	0.247^**^	0.023	10.552	−0.814^**^	0.104	−7.820	−0.909^**^	0.104	−8.745
Social activity				−0.383^**^	0.044	−8.761			
*R*^2^	0.129			0.407			0.402		
*p*	<0.001			<0.001			<0.001		

*p < 0.05, **p < 0.01.

**Table 5:** Results of mediation analysis.

Model	*c*	*a*	*b*	a*b	a*b95%CI	*c*’	Conclusion
Hearing impairment = > Social activity = > Cognitive decline	−1.2390^**^	−0.0336	−0.3835^**^	0.0129	−0.0056 to 0.0333	−1.2519^**^	Not significant
Functional mobility impairment = > Social activity = > Cognitive decline	−0.9086^**^	0.2470^**^	−0.3835^**^	−0.0947	−0.1228 to −0.0695	−0.8139^**^	Partial mediation

*c*, total effect; *c*’, direct effect, a*b, mediate effect. *p < 0.05, **p < 0.01.

## 4 Discussion

The findings of this study indicate that cognitive function in the older adult is significantly influenced by various factors, including gender, age, education level, residency, living arrangement, hyperlipidemia, cerebrovascular disease, alcohol consumption, smoking, ADLs, IADLs, as well as hearing, functional mobility, and social activity engagement. Collectively, these factors contribute to 40.22% of the observed cognitive decline. Moreover, social activity engagement was found to partially mediate the relationship between functional mobility impairment and cognitive decline. However, it did not significantly impact the relationship between self-reported hearing impairment and cognitive decline.

### 4.1 Risk factors for cognitive decline

The risk factors for cognitive decline in older adults identified in this study aligned closely with those reported in previous research. The 2020 Lancet Commission report highlighted 12 potentially modifiable risk factors for dementia, including lower education levels, hypertension, hearing impairment, smoking, obesity, depression, physical inactivity, diabetes, limited social contact, alcohol consumption, traumatic brain injury, and air pollution. Addressing these factors could potentially prevent or delay up to 40% of dementia cases (12). However, much of the evidence in that report was based on studies from high-income countries, suggesting possible variations across different cultural and environmental contexts. Consequently, this study incorporated significant risk factors identified here as control variables in the mediation analysis to assess the impact of social activity on hearing and functional mobility in relation to cognitive decline.

### 4.2 Relationship between social activity engagement and cognition

This study has established that social activity engagement serves as both an independent risk factor for cognitive decline and a mediator between functional mobility and cognitive status, aligning with prior research. A 28-year follow-up study in the UK involving 10,308 participants demonstrated that increased social contact at age 60 correlated with a reduced dementia risk over 15 years (HR for one standard deviation increase in social contact frequency 0.9, 95% CI 0.8–1.0) (35). Similarly, a Japanese longitudinal cohort study with 13,984 adults aged over 65, spanning an average of 10 years, employed a five-point social contact scale encompassing marital status, family support exchange, friend contact, community group participation, and paid work. This study found a linear association between higher scores on the scale and decreased dementia risk, with individuals scoring highest being 46% less likely to develop dementia compared to those with the lowest scores (36). Beyond dementia risk, social contact also directly influenced other aspects of mental and physical health, including mortality rates. Research indicated that stronger social connections were linked to improved health outcomes and increased longevity (37, 38). These findings collectively suggest that enhanced social contact in late middle age could modestly reduce dementia risk, even after adjusting for socio-economic and other lifestyle factors.

### 4.3 Relationship among hearing impairment, social activity, and cognitive decline

Hearing loss currently affects approximately 20% of the global population, which equates to over 1.5 billion people (39). It is a prominent and extensively researched risk factor for cognitive decline. Consistent with previous findings, our study confirmed the significant association between self-reported hearing impairment and cognitive decline. However, the impact of hearing on cognition might extend beyond social activity engagement. Prior research has shown that hearing loss can increase social isolation and loneliness in older adults, often due to challenges in communication and engagement, resulting in reduced participation in social activities (40, 41). The connection between social isolation or loneliness and dementia is well-documented (17). Thus, hearing impairment may lead to decreased social interaction, which in turn can contribute to cognitive impairment (15, 42). Our study presents findings that diverge from some established hypotheses regarding the relationship between hearing impairment and cognitive decline, particularly the cascade hypothesis which posits that hearing impairment decreases cognitive levels by reducing social activity. Notably, only 19.35% of our participants reported hearing impairment, a figure significantly lower than the prevalences documented in other epidemiological studies (43, 44). This discrepancy might be due to the reliance on self-reported data for defining hearing impairment in our study, which could differ from objective measurements like audiometry. Cultural aspects related to aging and attitudes toward well-being in late adulthood, particularly in China, might also influence these findings. With its dense older adult population, close-knit family structures, collectivist culture, isolation and loneliness among older Chinese adults is potentially lower than other countries (45, 46), which diminishing the impact of hearing impairment on socialization. Given these factors, this study do not support the cascade hypothesis, as we did not observe a direct pathway from hearing impairment to cognitive decline mediated by decreased social activity. The relationship between auditory and cognitive function may be more complex and influenced by various confounding factors.

### 4.4 Relationship among functional mobility, social activity, and cognitive decline

Physical function impairment is a common issue among older adults, with estimates suggesting that up to 50% of individuals over 80 years old may experience some form of motor impairment (47). Research has indicated that physical impairments can both precede and be predictive of the onset of mild cognitive impairment (48). However, these studies often focus on adults with specific mobility dysfunctions or motor disabilities, frequently linked to neurological conditions like Alzheimer’s or Parkinson’s disease (21, 49). Such conditions can disrupt social connectedness due to challenges such as limited transportation or inaccessible physical environments (50), which may not accurately represent the broader older adult population. In this study, we evaluated functional mobility as a variable to investigate its relationship with cognitive performance in the older adult.

Functional mobility refers to an individual’s physiological capacity to move independently and safely in various environments, essential for performing daily activities and maintaining participation in daily life (51). Impaired functional mobility was associated with increased risks of falls, loss of independence, and institutionalization (52). The Timed Up-and-Go (TUG) test, an objective tool for assessing basic functional mobility, was utilized in our analysis (53). The results indicated that functional mobility was an independent risk factor for cognitive decline in older adults. Moreover, older adults with impaired functional mobility and lower levels of social activity were more likely to experience cognitive decline compared to their more socially active counterparts. This finding aligned with other studies focusing on the impact of physical performance on cognition (54, 55). Higher levels of functional mobility are linked to a lower likelihood of cognitive decline. The mediating role of social activity engagement is significant, as older adult individuals with limited mobility often avoid social activities, leading to reduced cognitive stimulation and potentially contributing to cognitive decline.

The study’s findings shed light on the mechanisms of cognitive decline in older adults, emphasizing the mediating role of social activity engagement between functional mobility impairment and cognitive deterioration. This highlights the complexity of cognitive aging and the need for tailored interventions. Promoting social engagement through public initiatives, such as community activities and volunteer programs, could be an effective strategy to delay cognitive decline in older adults, especially for older adult with mobility impairment, as these interventions might foster cognitive resilience by offering mental stimulation, emotional support, and a sense of purpose. This is significant for improving the quality of life for older adults and reducing the caregiving burden on both families and the nation.

### 4.5 Strengths and limitations

Our study boasts significant strengths, such as its large sample size of older adults and an exhaustive baseline survey, allowing for the adjustment of numerous pertinent confounding factors. Nonetheless, it is important to recognize its limitations. The use of a cross-sectional study design constrains our capacity to establish causal relationships between the variables. Furthermore, the reliance on self-reported data for hearing impairment could lead to measurement bias and may not align with objective assessments. Additionally, employing convenience sampling for participant recruitment might have introduced selection bias, favoring respondents who were more accessible or more inclined to engage in the study. Lastly, the study was conducted in an eastern province of China. While the healthcare institutions were selected randomly, geographic and cultural differences could influence the results.

## 5 Conclusion

Our study determined that factors such as age, education level, residency, living arrangement, hyperlipidemia, cerebrovascular disease, alcohol consumption, smoking, ADLs, IADLs, as well as hearing, functional mobility, and social activity engagement, were associated with cognitive decline in Chinese older adults. Notably, social activity engagement emerged as a mediating factor in the link between functional mobility impairment and cognitive decline, but it did not mediate the relationship between self-reported hearing impairment and cognitive decline. These results underscored the importance of social activity engagement in potentially preventing cognitive decline in older adults with functional mobility impairments. The study emphasized the need to promote social engagement as a viable approach to mitigate cognitive deterioration in this demographic.

## Data Availability

The raw data supporting the conclusions of this article will be made available by the authors, without undue reservation.
